# *Lactobacillus casei Zhang* Counteracts Blood-Milk Barrier Disruption and Moderates the Inflammatory Response in *Escherichia coli*-Induced Mastitis

**DOI:** 10.3389/fmicb.2021.675492

**Published:** 2021-06-24

**Authors:** Yuhui Zheng, Gang Liu, Wei Wang, Yajing Wang, Zhijun Cao, Hongjian Yang, Shengli Li

**Affiliations:** State Key Laboratory of Animal Nutrition, College of Animal Science and Technology, China Agricultural University, Beijing, China

**Keywords:** *Lactobacillus casei* Zhang, bovine mammary epithelial cells, blood-milk barrier, mastitis, inflammatory response

## Abstract

*Escherichia coli* is a common mastitis-causing pathogen that can disrupt the blood-milk barrier of mammals. Although *Lactobacillus casei* Zhang (LCZ) can alleviate mice mastitis, whether it has a prophylactic effect on *E. coli*-induced mastitis through intramammary infusion, as well as its underlying mechanism, remains unclear. In this study, *E. coli*-induced injury models of bovine mammary epithelial cells (BMECs) and mice in lactation were used to fill this research gap. *In vitro* tests of BMECs revealed that LCZ significantly inhibited the *E. coli* adhesion (*p* < 0.01); reduced the cell desmosome damage; increased the expression of the tight junction proteins claudin-1, claudin-4, occludin, and zonula occludens-1 (ZO-1; *p* < 0.01); and decreased the expression of the inflammatory cytokines tumor necrosis factor (TNF)-α, interleukin (IL)-1β, and IL-6 (*p* < 0.01), thereby increasing trans-epithelial electric resistance (*p* < 0.01) and attenuating the lactate dehydrogenase release induced by *E. coli* (*p* < 0.01). *In vivo* tests indicated that LCZ significantly reduced the injury and histological score of mice mammary tissues in *E. coli*-induced mastitis (*p* < 0.01) by significantly promoting the expression of the tight junction proteins claudin-3, occludin, and ZO-1 (*p* < 0.01), which ameliorated blood-milk barrier disruption, and decreasing the expression of the inflammatory cytokines (TNF-α, IL-1β, and IL-6) in mice mammary tissue (*p* < 0.01). Our study suggested that LCZ counteracted the disrupted blood-milk barrier and moderated the inflammatory response in *E. coli*-induced injury models, indicating that LCZ can ameliorate the injury of mammary tissue in mastitis.

## Introduction

Bovine mastitis is a common disease that results in enormous economic losses for dairy farms worldwide (Sathiyabarathi et al., 2016; Angelopoulou et al., 2018). *Escherichia coli* (*E. coli*) is one of the most common mastitis-causing pathogens and often causes acute mastitis with a severe inflammatory response that disturbs the blood-milk barrier. The abuse of antimicrobials in food animals has attracted public attention, and concerns relating to antimicrobial residues and anti-microbial resistance in bacteria have increased because of the increased use of antimicrobials (Lhermie et al., 2016; Muziasari et al., 2016). There is thus an urgent need to find new potential non-antimicrobials to prevent mastitis.

*Lactobacillus* is an important probiotic that contributes to the immunity and health of animals. Their probiotic properties play an important role in regulating gut microbiota (Igor et al., 2019), relieving intestinal diseases (Sanders et al., 2019), and enhancing body immunity (Garcia-Castilla et al., 2019) in addition to other functions (da Costa et al., 2016; Li et al., 2017). *Lactobacillus* is known to protect hosts against intramammary infection in both humans and cows by oral or intramammary administration (Jiménez et al., 2008; Arroyo et al., 2010; Fernández et al., 2016), but the mechanism of this protective effect remains unclear. Intramammary infusion of drugs is the most common and effective way for treating bovine mastitis in practice, and intramammary infusion of some *Lactobacillus* strains can effectively alleviate clinical symptoms of bovine mastitis (Ignacio et al., 2015; Pellegrino et al., 2017; Kitching et al., 2019). The clinical symptoms of mastitis in mice can be effectively alleviated through oral administration of *Lactobacillus casei* Zhang (LCZ; Ma et al., 2018). However, whether intramammary infusion of LCZ has a prophylactic effect on mastitis, as well as its underlying mechanism, remains unclear. Here, we studied the prophylactic effect of LCZ on *E. coli*-induced injury models and its mechanism using bovine mammary epithelial cells (BMECs) and mice in lactation.

## Materials and Methods

### Bacterial Strains

*Lactobacillus casei* Zhang (provided by Inner Mongolia Agricultural University, Key Laboratory of Dairy Biotechnology and Engineering, Inner Mongolia, China) was grown in de Man, Rogosa, and Sharpe (MRS) broth (Solarbio, Beijing, China) at 37°C under microaerophilic conditions. The isolate of *E. coli* ATCC 25922 (provided by China General Microbiological Culture Collection Center, Beijing, China) was incubated in tryptone soya broth (Solarbio, Beijing, China) at 37°C. In all treatments, *E. coli* and LCZ were plated on tryptone soya agar (TSA, Solarbio, Beijing, China) and MRS agar (Solarbio, Beijing, China), respectively, to count colony-forming unit (CFU).

### Cell Culture

Bovine mammary epithelial cells (provided by Yangzhou University, College of Animal Science and Technology Feed Engineering Technology Research Center, Yangzhou, China) were cultivated in Dulbecco’s modified eagle medium (DMEM)-F12 medium (ThermoFisher, Waltham, MA, United States) containing 10% fetal bovine serum (FBS; ThermoFisher), 1% penicillin and streptomycin (Beyotime, Shanghai, China), 15 ng/ml epidermal growth factor (PeproTech, Cranbury, NJ, United States), 1% non-essential amino acids (ThermoFisher), and 1% insulin-transferrin-selenium (ITS, ThermoFisher) at 37°C in 5% CO_2_ and 95% relative humidity. Briefly, BMECs (5 × 10^5^) were seeded onto six-well cell culture plates in the aforementioned medium for 48 h and were then cultured in DMEM-F12 medium with 5% FBS, 1% penicillin and streptomycin, and 1% ITS for 72 h to ensure that a polarized and differentiated state was achieved. Before treatments, the medium was changed into DMEM-F12 alone, and both *E. coli* and LCZ were diluted by DMEM-F12. Cells were randomly treated under four conditions, and the dose of two bacteria was decided according to pretest: (1) Control group (DMEM alone); (2) 1 × 10^3^ CFU *E. coli* treatment for 5 h (*E. coli* group); (3) 1 × 10^5^ CFU LCZ pretreatment for 3 h, followed by 1 × 10^3^ CFU *E. coli* treatment for 5 h (LCZ + *E. coli* group); and (4) 1 × 10^5^ CFU LCZ treatment for 3 h (LCZ group). 5 h after LCZ treatment, cells were washed with phosphate buffer saline (PBS, ThermoFisher) and then incubated with *E. coli*. All cell experiments were repeated three times, and each experiment was performed in triplicate.

### Cell Death Assay

Cell death was measured using the lactate dehydrogenase (LDH) method through a CytoTox 96 cytotoxicity assay (Solarbio, Beijing, China) based on the manufacturer’s manual. In detail, the cell culture supernatant was used to detect the amount of LDH release.

### Adhesion Assay

The BMECs were washed with PBS for three times, and 1 ml triton-X-100 (0.5% v/v, Solarbio, Beijing, China) was added to each well after incubation with LCZ or *E. coli*. The cells were scraped off, and the lysate was transferred to a centrifugal tube after 10 min of stabilization at room temperature. *E. coli* and LCZ were plated on TSA and MRS agar, respectively, to count CFU.

### Trans-Epithelial Electric Resistance

Bovine mammary epithelial cells were seeded on transwell inserts (membrane area: 0.33 cm^2^, pore size: 0.4 μm, Corning, NY, United States), cultivated for 120 h as described previously, and measured TEER daily. When cells reached a polarized and differentiated state which means that TEER reached 400 Ω cm^2^, different treatments were applied. TEER was measured for three times per well using a Millicell Electrical Resistance System-2 (Millipore, MA, United States) and expressed as Ω cm^2^ after subtracting the filter resistance value. The results of TEER were defined as the TEER change (TEER of monolayer cells after treatments – TEER of monolayer cells before treatments).

### Animals

CD-1 mice (8–10 days in lactation; 50 ± 5 g; obtained from SPF Biotechnology Co., Ltd., Beijing, China) were reared at 25°C and fed with enough food and water. Twenty mice were randomly allocated to the four treatments, and the dose of two bacteria was decided according to pretest: (1) Control group (PBS alone); (2) 1 × 10^3^ CFU *E. coli* treatment for 12 h (*E. coli* group); (3) 1 × 10^5^ CFU LCZ pre-infusion for 24 h, followed by 1 × 10^3^ CFU *E. coli* treatment for 12 h (LCZ + *E. coli* group); and (4) 1 × 10^5^ CFU LCZ treatment for 24 h (LCZ group). Five mice were used as replicates in each group. Mice were anesthetized with Zoletil 50 (Virbac, Barneveld, France), and all treatments were conducted through intramammary infusion *via* the teat canal of the fourth teat. *E. coli* was injected 24 h after LCZ pretreatment. After treatments, the mice were killed to collect mammary tissues. All animal feeding and experimental protocols in this study were approved by the animal ethics committee of the National Institutes of Health Guide for the Care and Use of Laboratory Animals (GB14925-2010, China).

### Histopathology

The general condition of each mammary tissue of the mice was assessed by clinical scoring. The clinical score ranked from 1 to 5, with higher scores corresponding to greater degrees of tissue damage. Specifically, 1 indicated no damage, 2 indicated slight redness, 3 indicated redness and slight bleeding, 4 indicated redness and bleeding, and 5 indicated redness and obvious bleeding.

The mammary tissues were fixed in 4% cell tissue fixative (Solarbio) immediately after the mice were killed. To evaluate histological changes, hematoxylin-eosin was used to stain tissue samples, and a microscope was used to observe the tissues. The semi-quantitative scoring was performed using a histological score based on the following criteria. The histological score ranged from 1 to 5, and higher scores corresponded to greater degrees of tissue damage. Specifically, 1 indicated the absence of histological features (i.e., necrosis, neutrophils, and lymphocytes), 2 indicated minimal histological features (i.e., individual neutrophils), 3 indicated mild histological features (i.e., a small amount of neutrophils), 4 indicated moderate histological characteristics (i.e., many neutrophils and slight damage to glandular structure), and 5 indicated severe histological characteristics (i.e., a large number of neutrophils and severe damage to glandular structure). Both clinical scoring and histological scoring were made by experienced veterinary pathologists who were blinded to the treatments.

### Immunofluorescence

Two mammary tissue blocks (0.3 cm^3^) were immediately sampled after the mice were killed and then stored in liquid nitrogen for tissue immunofluorescence to localized tight junction proteins. Briefly, cryosections of mammary tissues were fixed with 4% paraformaldehyde and then were incubated with 5% bovine serum albumin (BSA; ThermoFisher) at 25°C. After incubation using primary antibodies (zonula occludins-1 (ZO-1), claudin-3, and occludin, 1:2,000 dilution, Abcam, Cambridge, United Kingdom) at 4°C for 12 h, tissues were washed with PBS, and secondary antibodies (1:1,000 dilution, CoWin Biosciences, Beijing, China) were added and incubated at 25°C for 1 h. Blanks were treated in the same way except that the tissues were not incubated with primary antibodies. After washing with PBS and staining with 4′,6-diamidino-2-phenylindole (DAPI), a fluorescence microscope was used to obtain images.

### Transmission Electron Microscope Analysis

Two mammary gland tissue blocks (10 mm^3^) collected from each mouse were immediately stored in centrifuge tubes and filled with electron microscope fixative (Servicebio, Wuhan, China) for transmission electron microscopy (TEM; Gang et al., 2019). Cell samples were harvested and fixed with electron microscope fixative for TEM. Both tissue and cell samples were dehydrated with ethanol at 25°C for 15 min per step. Cells were then placed into epoxy resin acetone mixtures (Solarbio) for 2 h and then into pure resin (Solarbio) overnight at 37°C. Next, an ultramicrotome (Leica EM, Wetzlar, Germany) was used to cut ultra-thin sections, which were stained by uranyl acetate and lead citrate (1%, Solarbio) and detected using TEM (Hitachi H-7650, Tokyo, Japan).

### Western Blot Analysis

Both BMECs and mammary tissues were collected after treatment, and protein content was determined using a BCA assay kit (Beyotime). Each sample was separated by 12% sodium dodecyl sulfate-polyacrylamide gels, transferred onto polyvinylidene fluoride membranes (Bio-Rad, CA, Hercules, United States), and blocked by 5% BSA for 12 h at 4°C. The membranes were cultured with primary antibodies (zonula occludins-1 (ZO-1), claudin-1, claudin-4, and occludin for BMECs, 1:500 dilution, Bioss Antibodies, Beijing, China; ZO-1, claudin-3, and occludin for mice mammary tissue, 1:2,000 dilution, Abcam, Cambridge, United Kingdom) at 25°C for 3 h. After washing with Tris-buffered saline with tween (NobleRyder, Beijing, China), the membranes were incubated with secondary antibodies (1:1,000 dilution, CoWin Biosciences) at 25°C for 1 h. Protein bands were visualized by a Beyo Enhanced Chemiluminescence reagent kit (Solarbio). MultiImager (Bio-Rad Gel Doc XR) and ImageJ software (National Institutes of Health, Bethesda, MD, United States) were used to detect protein expression.

### Enzyme-Linked Immunosorbent Assay

Tumor necrosis factor (TNF)-α, IL-6, and IL-1β expression in both BMECs and mammary tissues of mice were detected using bovine (DG Biotech, Beijing, China) and mouse ELISA kits (ThermoFisher), respectively. In detail, the cell medium was moved to a sterile centrifuge tube and centrifuged at 4°C for 10 min at 1000 × g for further test. Each tissue sample was added with 2 ml of RIPA tissue lysate (Solarbio), the cracked samples were centrifuged at 10,000–14,000 g for 4 min, and the supernatant was removed for further test.

### Statistical Analysis

All data were analyzed in SAS 9.2 (SAS Institute Inc., NYC, United States) and expressed as mean ± standard error of the mean (SEM). Graphs were produced with Origin 8.0 (Origin Lab, Northampton, MA, United States). Variance was analyzed using one-way ANOVA in SAS 9.2, followed by multiple comparisons using the Tukey method. The threshold for statistical significance was *p* < 0.01.

## Results

### *In vitro* Pre-incubation With LCZ Reduces *E. coli*-Induced Damage of BMECs

**Figure 1:**
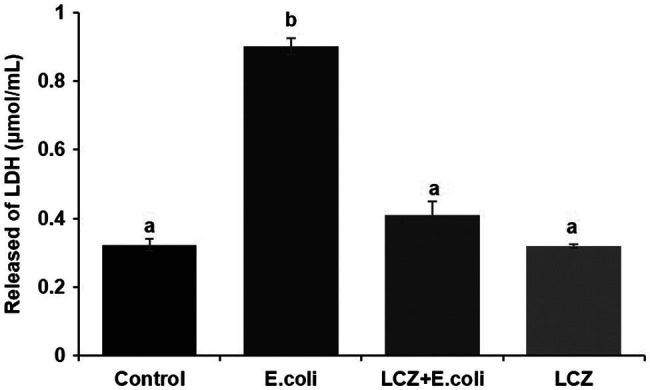
Effect of *Lactobacillus casei* Zhang (LCZ) on changes in cell viability (*n* = 9). Cell viability was determined by the LDH method. Means with different superscripts differ significantly (*p* < 0.01).

**Figure 2:**
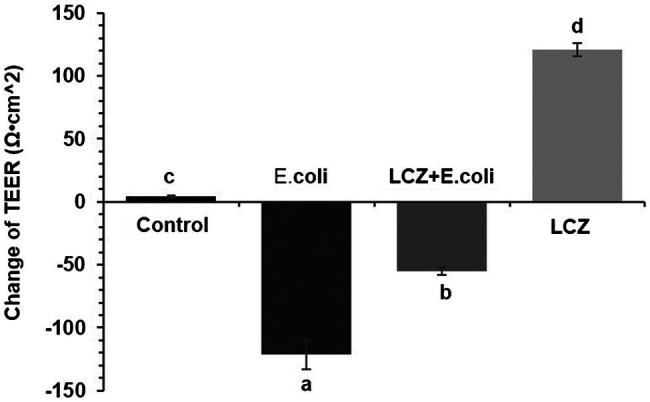
Effect of LCZ on changes in TEER (*n* = 9). TEER was obtained using a Millicell Electrical Resistance System-2. TEER change = TEER of monolayer cells after treatment – TEER of monolayer cells before treatment. Means with different superscripts differ significantly (*p* < 0.01).

**Figure 3:**
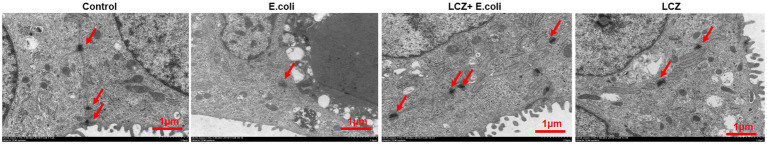
Effect of LCZ on changes in blood-milk barrier structure (*n* = 9). Desmosome structure was detected using transmission electron microscopy (TEM). Scale bars: 1 μm. Means with different superscripts differ significantly (*p* < 0.01).

*Escherichia coli* caused a significantly higher LDH release compared with the other groups (*p* < 0.01); there were no significant differences between the Control, LCZ + *E. coli*, and LCZ groups (Figure 1). TEER change in BMECs treated with *E. coli* was negative and significantly different from the other groups (*p* < 0.01). Specifically, although TEER change was significantly higher in the LCZ + *E. coli* group than in the Control group (*p* < 0.01), it was significantly less negative compared with the *E. coli* group (*p* < 0.01; Figure 2). The desmosome structure in the *E. coli* group was severely damaged and blurred as illustrated by TEM, whereas LCZ pretreatment noticeably alleviated desmosome structure damage. Moreover, the desmosome structure in the LCZ group was clearly visible, and no clear difference was detected compared with the Control group (Figure 3).

**Figure 4:**
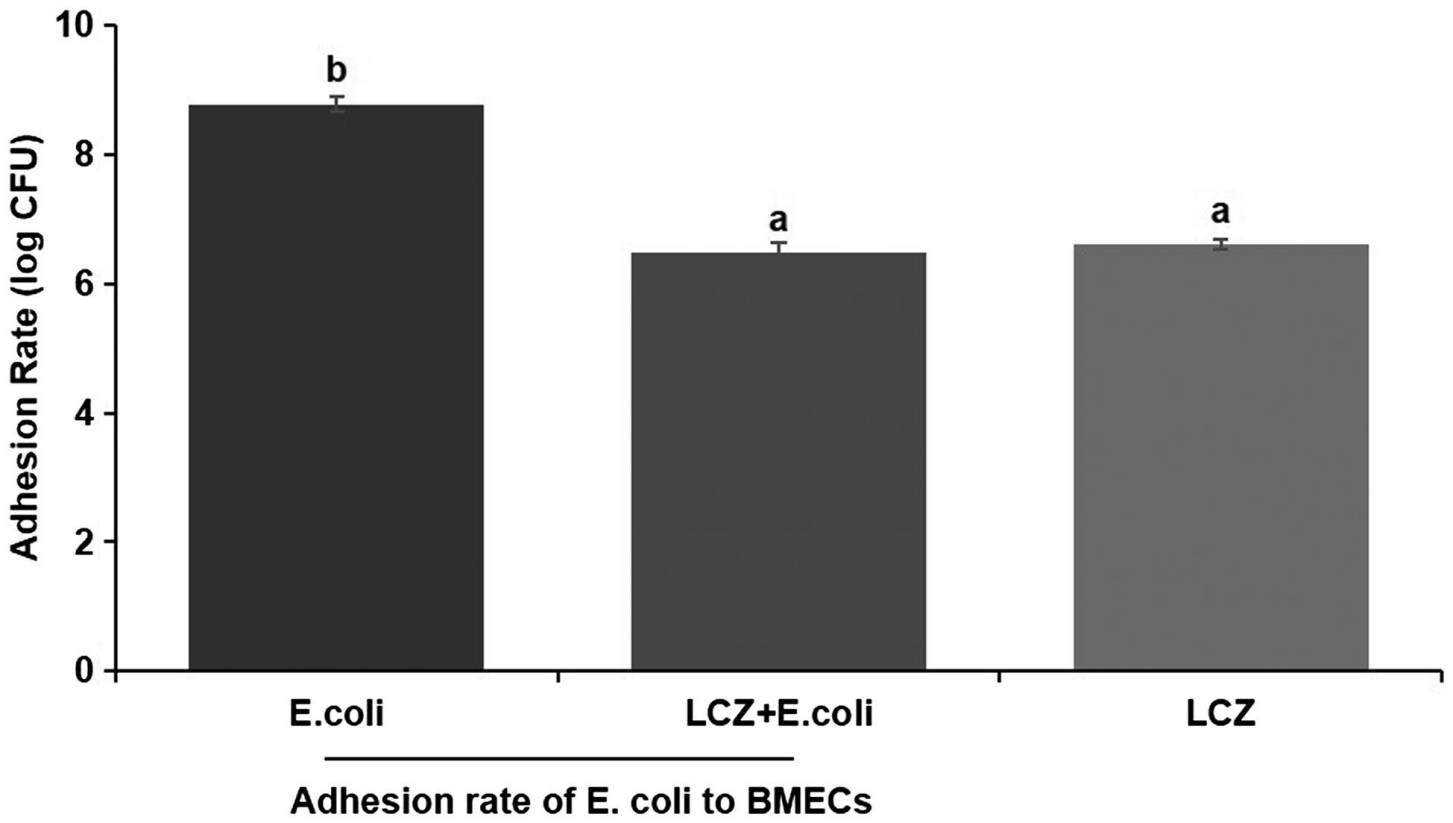
Effect of LCZ on the adhesion rate of *E. coli* to bovine mammary epithelial cells (BMECs; *n* = 9). Means with different superscripts differ significantly (*p* < 0.01).

**Figure 5:**
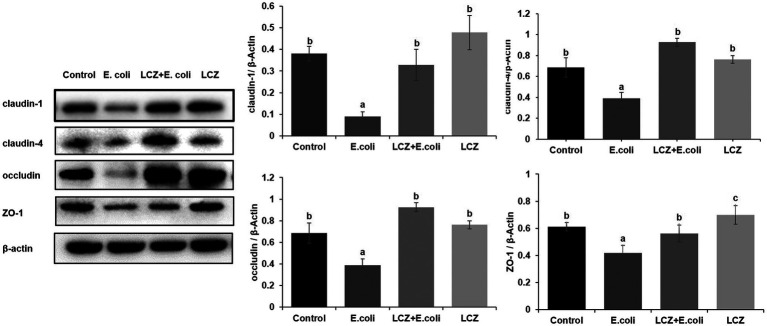
Effect of LCZ on the tight junction protein expression of BMECs (*n* = 9). Quantification of proteins was normalized to β-actin. Means with different superscripts differ significantly (*p* < 0.01).

**Figure 6:**
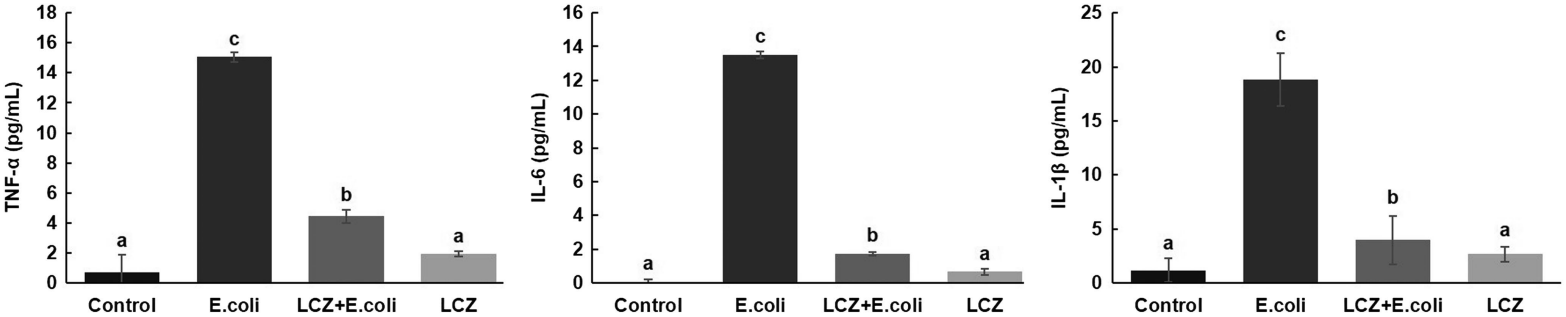
Effect of LCZ on the inflammatory cytokine transcription of BMECs (*n* = 9). TNF-α, IL-1β, and IL-6 transcriptions were measured by ELISA. Means with different superscripts differ significantly (*p* < 0.01).

LCZ pretreatment significantly inhibited the *E. coli* adhesion rate to monolayer BMECs (*p* < 0.01), and the adhesion ability of LCZ to monolayer BMECs was significantly lower compared with *E. coli* (*p* < 0.01; Figure 4). The expression of claudin-1, claudin-4, occludin, and ZO-1 in BMECs was significantly decreased in the *E. coli* group compared with the other groups (*p* = 0.02; Figure 5), and LCZ pretreatment significantly increased the expression of these four proteins (*p* = 0.02). Moreover, ZO-1 expression in the LCZ group was significantly increased (*p* = 0.02); there were no significant differences in the expression of the other three proteins between the LCZ group and the Control group. The effect of LCZ on the expression of inflammatory cytokines in BMECs is shown in Figure 6. The expression of TNF-α, IL-6, and IL-β was significantly higher in the *E. coli* group than in the other groups (*p* < 0.01); LCZ pretreatment inhibited the expression of these cytokines (*p* < 0.01).

### *In vivo* Pre-infusion With LCZ Reduces *E. coli*-Induced Injury in Mice Mammary Tissue

**Figure 7:**
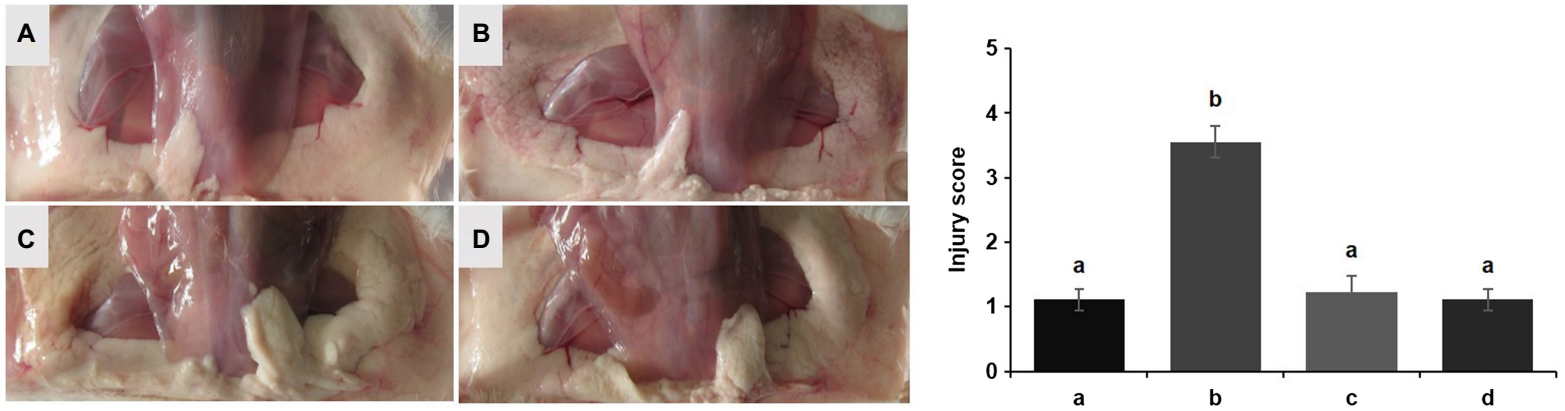
Effect of LCZ on the histopathological impairment of mice mammary tissue. Injury score of mice mammary tissue from the Control **(A)**, *E. coli* **(B)**, LCZ + *E. coli* **(C)**, and LCZ **(D)** groups. The injury score ranged from 1 to 5, where 1 indicates no damage, 2 indicates slight redness, 3 indicates redness and slight bleeding, 4 indicates redness and bleeding, and 5 indicates redness and obvious bleeding. Data shown are means ± standard error of the mean (SEM) (*n* = 10). Means with different superscripts differ significantly (*p* < 0.01).

**Figure 8:**
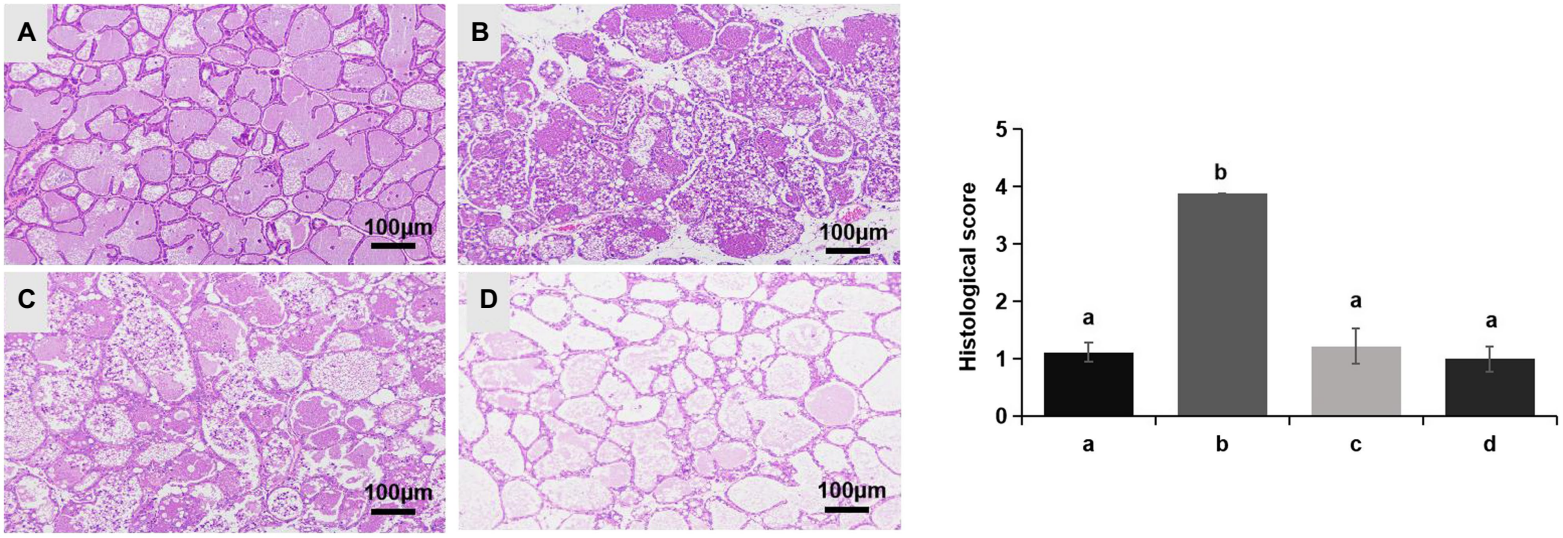
Effect of LCZ on the histopathological impairment of mice mammary tissue. Histological score of hematoxylin-eosin-stained pathological sections from the Control **(A)**, *E. coli* **(B)**, LCZ + *E. coli* **(C)**, and LCZ **(D)** groups. Hematoxylin-eosin staining of formalin-fixed mammary gland. Scale bars: 100 μm. The histological score ranged from 1 to 5, where 1 indicates the absence of histological features (e.g., necrosis, neutrophils, and lymphocytes), 2 indicates minimal histological features (i.e., individual neutrophils), 3 indicates mild histological features (i.e., a small amount of neutrophils), 4 indicates moderate histological characteristics (i.e., many neutrophils and slight damage to glandular structure), and 5 indicates severe histological characteristics (i.e., a large number of neutrophils and severe damage to glandular structure). Data shown are means ± SEM (*n* = 10). Means with different superscripts differ significantly (*p* < 0.01).

**Figure 9:**
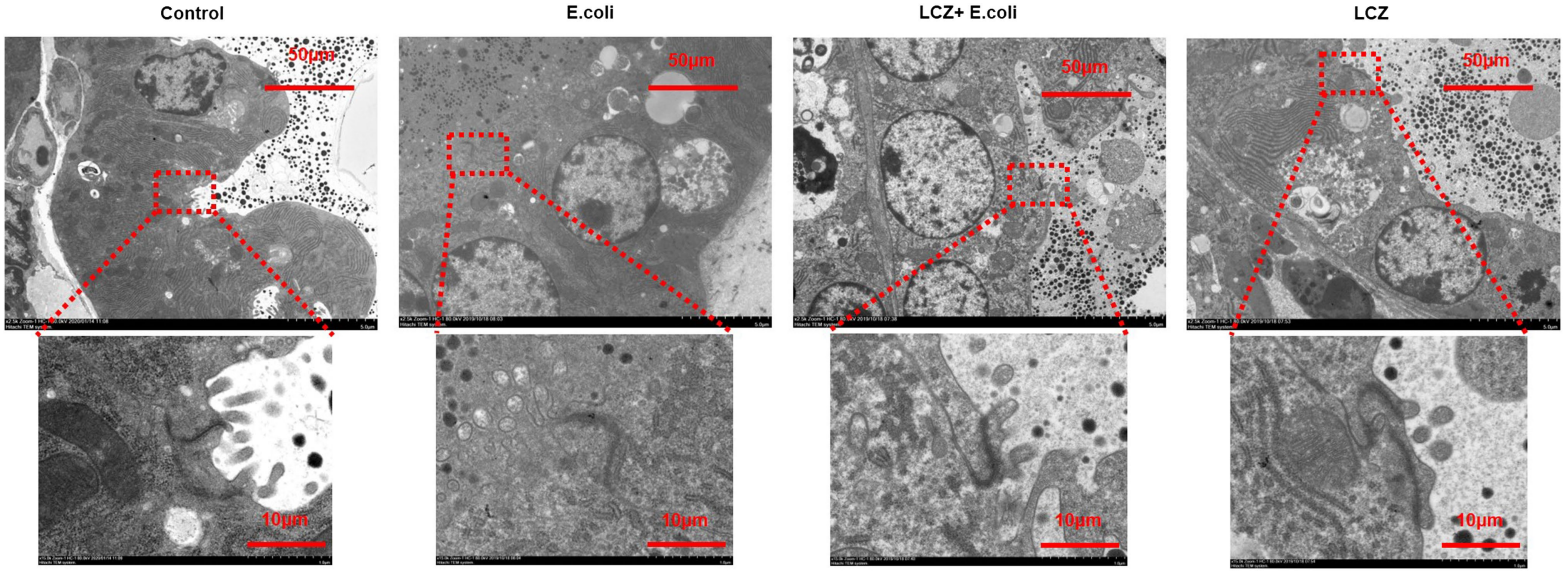
Effect of LCZ on the tight junction structure of mice mammary tissue. The structure of the tight junction was observed by TEM.

No redness or swelling was detected in the mammary tissues in the Control, LCZ + *E. coli*, and LCZ groups, and there was no significant difference in the injury score among these groups (Figure 7). However, visible redness and bleeding were observed in the *E. coli* group, and LCZ pretreatment significantly reduced the injury score (*p* < 0.01). No visible pathological damage was observed in the mammary acinar structure of the mice in the Control and LCZ groups; by contrast, *E. coli* caused remarkable pathological injury and led to the presence of a large number of neutrophils in the alveolar lumen (Figure 8). LCZ pretreatment significantly alleviated mammary acinar structure damage and the number of neutrophils compared with the *E. coli* group; furthermore, a significant decrease in the histological score was observed (*p* < 0.01). The tight junction structure in the *E. coli* group was severely damaged and blurred under TEM (Figure 9) but was visibly alleviated in the LCZ + *E. coli* group. The tight junction structure in the LCZ group was apparent, and there were no obvious differences between the LCZ group and the Control group.

**Figure 10:**
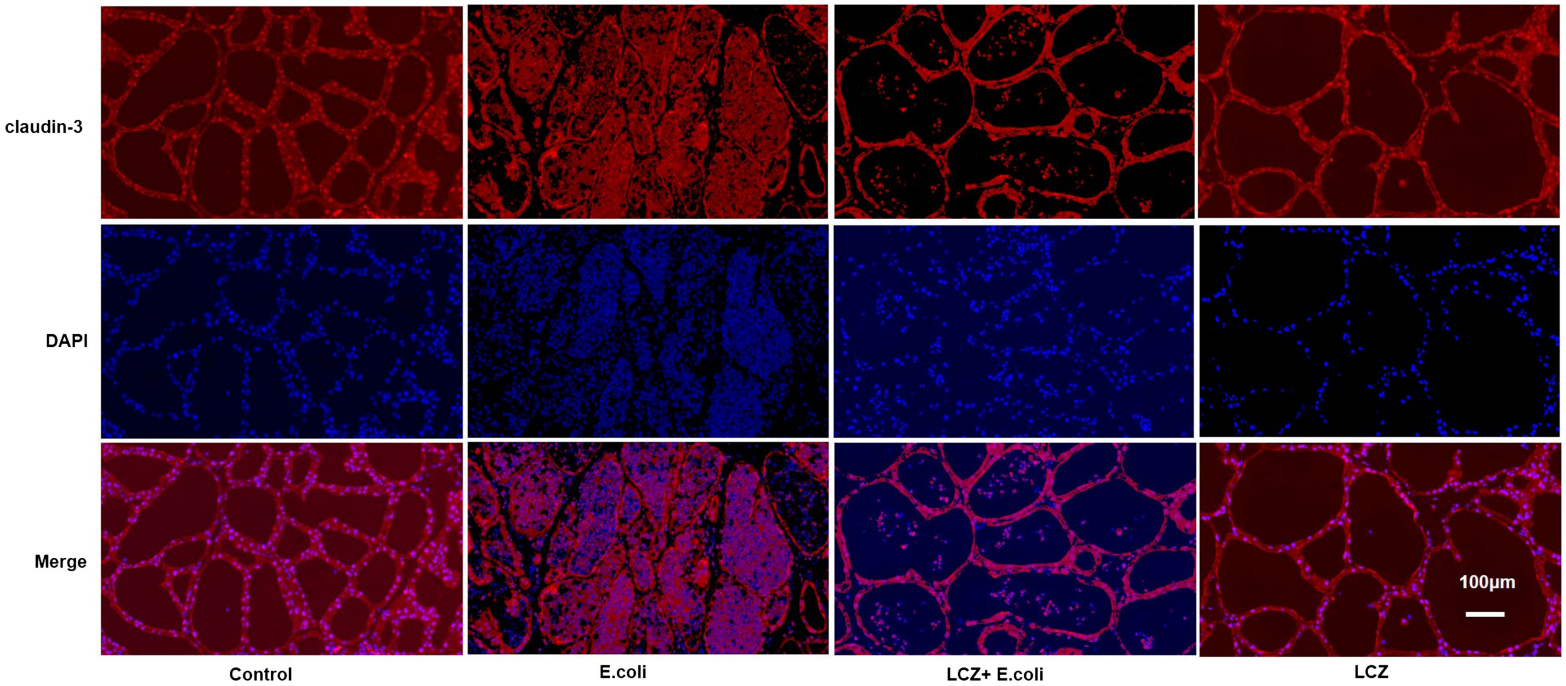
Effect of LCZ on the location of claudin-3 in mice mammary tissue. Blue shows nuclei (DAPI), and red shows claudin-3. Scale bars: 100 μm.

**Figure 11:**
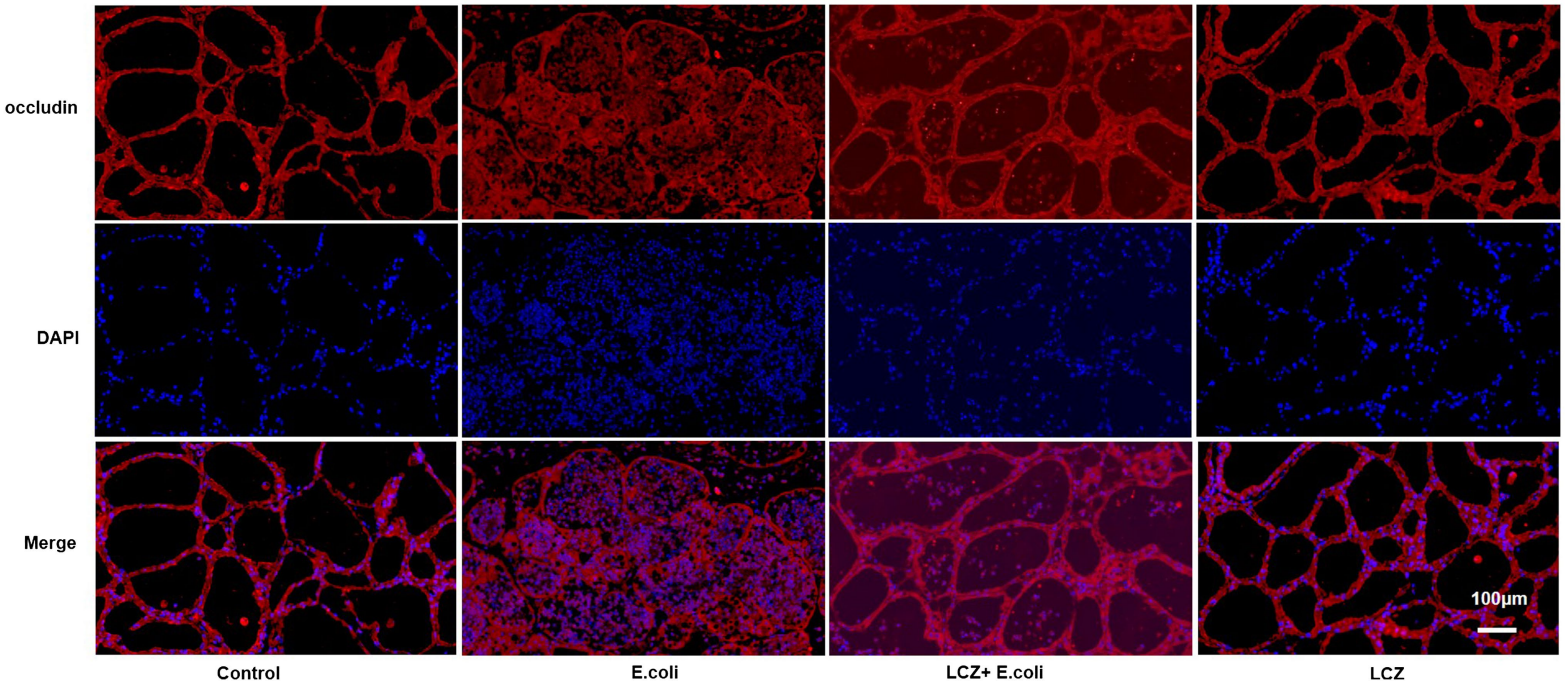
Effect of LCZ on the location of occludin. Blue shows nuclei (DAPI), and red shows occludin. Scale bars: 100 μm.

**Figure 12:**
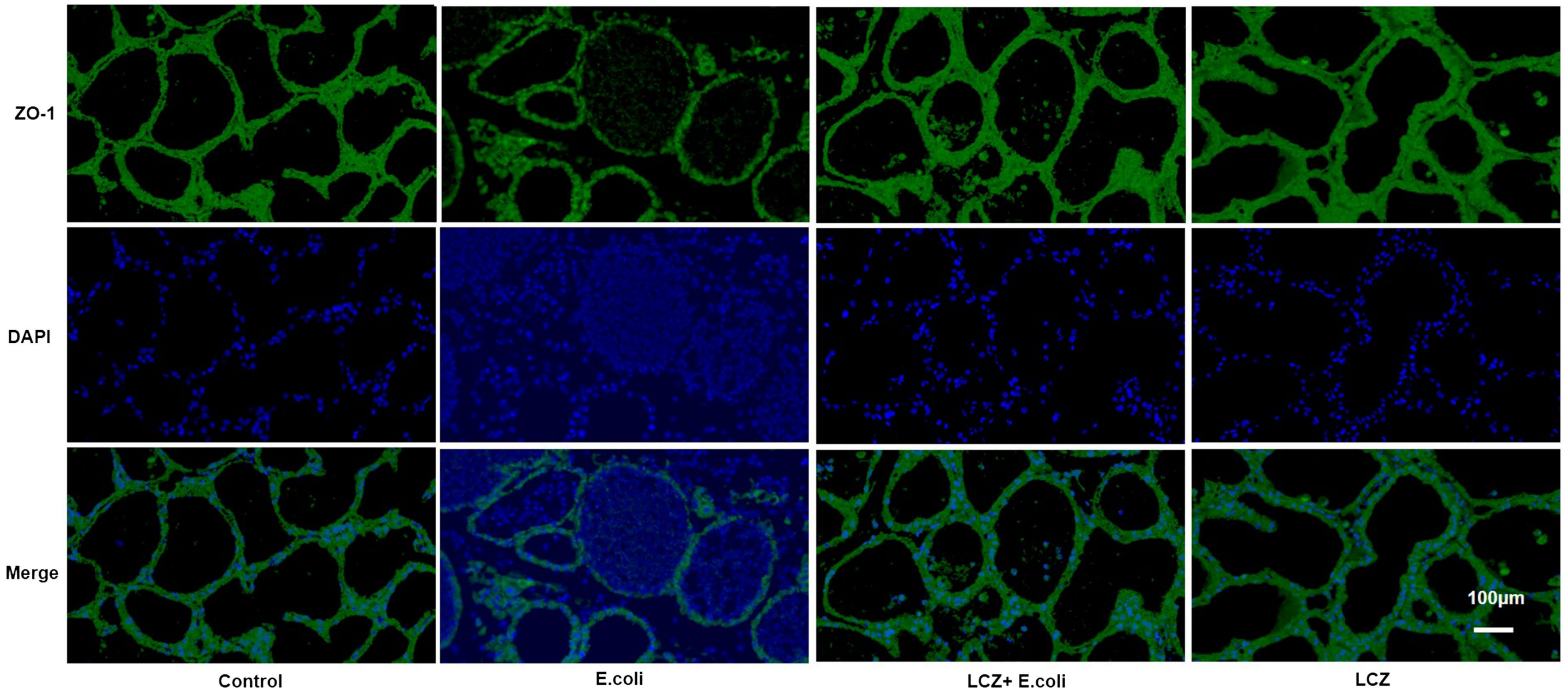
Effect of LCZ on the location of ZO-1 in mice mammary tissue. Blue shows nuclei (DAPI), and green shows ZO-1. Scale bars: 100 μm.

**Figure 13:**
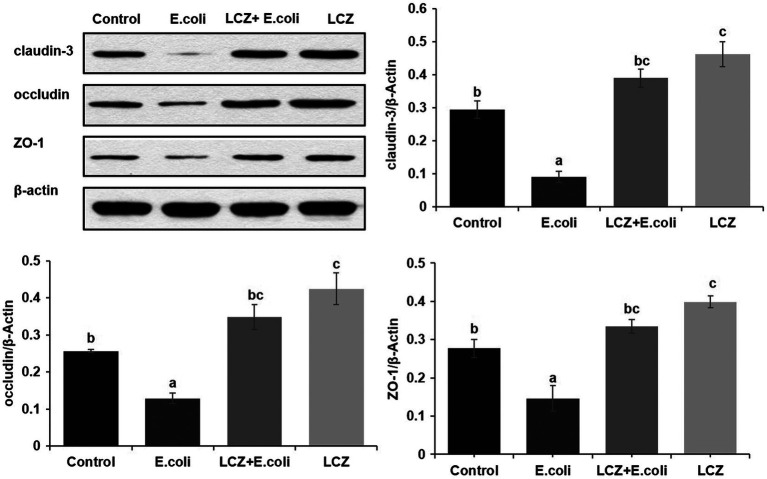
Effect of LCZ on the expression of claudin-3, occludin, and ZO-1 in mice mammary tissue. Quantification of proteins was normalized to β-actin. Data shown are means ± SEM (*n* = 10). Means with different superscripts differ significantly (*p* < 0.01).

**Figure 14:**
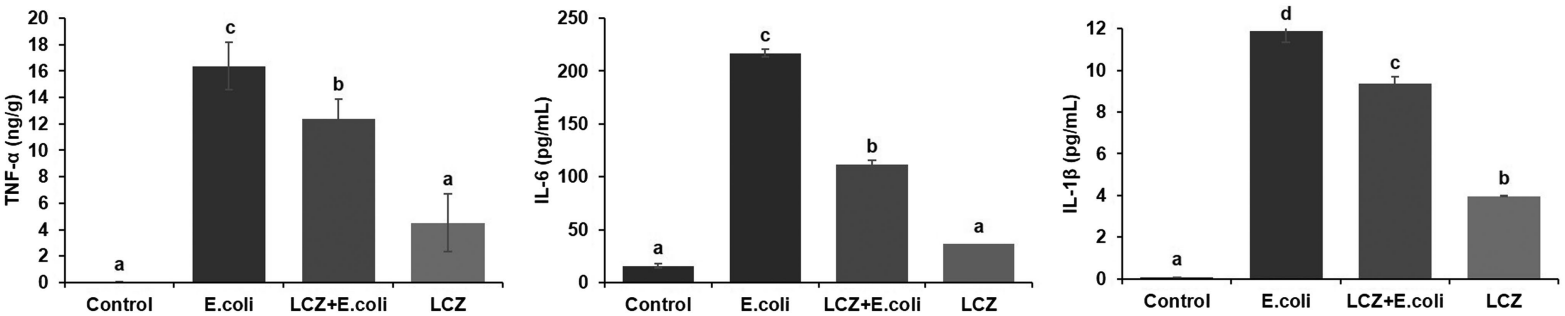
Effect of LCZ on the inflammatory cytokine transcription of mice mammary tissue (*n* = 10). Levels of TNF-α, IL-1β, and IL-6 were measured by ELISA. Means with different superscripts differ significantly (*p* < 0.01).

The tight junction proteins claudin-3, occludin, and ZO-1 in mice mammary tissue were clearly localized on the interstitial side, as well as in the intercellular regions of the neighboring alveolar epithelial cells before *E. coli* infusion (Control group); nevertheless, the localization of these three proteins was disturbed by *E. coli*. In addition, LCZ pretreatment greatly promoted the positive reactions of these proteins in the alveolar lumen compared with the *E. coli* group. These three proteins were all localized on the interstitial side, and no differences were noted between the LCZ and Control groups (Figures 10–12). Tight junction protein expression in mice mammary tissues was significantly decreased in the *E. coli* group relative to the other groups (*p* < 0.01; Figure 13). LCZ pretreatment significantly increased the expression of these proteins compared with the *E. coli* group (*p* < 0.01), and no significant differences were noted between the LCZ and Control groups. The effect of LCZ on inflammatory cytokine expression in mice mammary tissue is shown in Figure 14. TNF-α, IL-6, and IL-1β expressions were significantly increased by *E. coli* (*p* < 0.01), whereas LCZ pretreatment significantly decreased the expression of these cytokines (*p* < 0.01). IL-1β expression was significantly higher in the LCZ group than in the Control group (*p* < 0.01), and no significant differences were detected in IL-6 and TNF-α expressions.

## Discussion

*Lactobacillus casei* Zhang has a promising prophylactic effect against several inflammatory diseases (Wang et al., 2013, 2016; Zhang et al., 2017), and recent research has shown that LCZ can have a prophylactic effect against mastitis in mice when administered orally (Ma et al., 2018). Here, both *in vitro* and *in vivo* tests were conducted to characterize the prophylactic effect of LCZ on *E. coli*-stimulated BMECs and mice mammary injury; its potential mechanism was also studied. In dairy farming, the treatment of mastitis is mainly carried out through milk duct infusion; therefore, in this study, intramammary infusion instead of oral infusion was chosen during *in vivo* test. This study revealed that LCZ pretreatment could dramatically counteract BMEC injury induced by *E. coli*, a finding that is consistent with previous studies (Chen et al., 2017; Dinić et al., 2017; Wang et al., 2018a). The prophylactic effect of LCZ on *E. coli*-induced mastitis in mice by intramammary infusion was also explored in this study. The result showed that pre-infusion of LCZ could reduce the number of neutrophils and relieve damage to mammary tissue, which was also consistent with the results of *in vitro* test, which shows that LCZ pretreatment decreases the release of LDH of BMECs. Recent studies have shown that intramammary infusion of *Lactococcus* does not cause inflammatory reactions; after intramammary infusion of *Lactococcus*, immune proteins acute phase proteins haptoglobin and milk amyloid A were significantly expressed in the mammary glands of healthy Holstein cows (Pyorala, 2003; Eckersall et al., 2006; Crispie et al., 2008), and no major bovine mastitis pathogen was detected (Pellegrino et al., 2017). Therefore, findings of this study indicate that LCZ can prevent bovine mastitis, which might be related to its ability to promote the production of lactic acid, antibacterial peptides, and other beneficial substances (Lebeer et al., 2018).

Several studies have indicated that pro-inflammatory cytokines play an important regulatory role in the disorder of tight junctions induced by inflammation (Schulzke et al., 2009; Zhang et al., 2018). The integrity of the blood-milk barrier could protect mammary epithelial cells from infection by pathogens and thus prevent an inflammatory reaction. TNF-α and IL-1β disrupt tight junctions, and IL-6 is associated with host defense against inflammatory disease (Rochfort et al., 2014; Cheng et al., 2018). In this study, the expression of TNF-α, IL-6, and IL-1β caused by *E. coli* decrease significantly when pretreated with LCZ, which is consistent with the results of Wu et al. (2016) showing that *Lactobacillus* pretreatment effectively reduce the upregulation of TNF-α, IL-1β, and IL-8 mRNA expressions induced by *E. coli*. This may because that LCZ pretreatment may promote an early alert state of the immune system which could improve the alertness of the host defensive system, thus stopping a subsequent strong infection rapidly (Kingma et al., 2011). Furthermore, the previous studies have shown that the inflammatory response can decrease acinar tight junction protein expression (Hartwig et al., 2003; Oguro et al., 2011) and affect barrier function. *In vitro* test showed that LCZ could significantly inhibit inflammatory cytokine expression by inhibiting *E. coli* infection; we thus also measured inflammatory cytokine expression in mice mammary tissue and got the same result. Therefore, this study indicates that the prophylactic effect of LCZ may be achieved by increase the alert state increasing a little bit the levels of immune regulators, which allows to decrease the expression of inflammatory cytokines in both BMECs and mice mammary tissue caused by *E. coli*. Besides, in exploring the underlying mechanism of the prophylactic effect of LCZ against *E. coli*-treated cells and blood-milk barrier damage, this study found that LCZ pretreatment could significantly reduce the adhesion rate of *E. coli* to BMECs. These results are consistent with recent studies showing that *Lactobacillus* could significantly inhibit the adhesion of *E. coli* to Caco-2 (Behbahani et al., 2019) and HT-29 cells (Dhanani and Bagchi, 2013). Therefore, the prophylactic effect of LCZ on BMECs could be mediated by the inhibition of *E. coli* adhesion, so that the expression of cytokines was decreased lower, which lead to *E. coli* infection decrease.

Mammary epithelial cells form the blood-milk barrier through special connecting structures, such as tight junctions. Several mammary pathogenic bacteria, including *E. coli*, cause mastitis. The blood-milk barrier is leaky in bovine mastitis, which permits molecules to cross the barrier and occur in milk (Nguyen and Neville, 1998; Lehmann et al., 2013). Specifically, *E. coli* can damage tight junction structure, thereby increasing the permeability of the epithelial barrier and causing pathogens in the acinus to enter mammary tissue (Bruewer et al., 2006; Shen and Turner, 2006). TEER reflects the integrity of the monolayer cells, the ion conductance of the pathway adjacent to monolayer epithelial cells, and the pore size of tight junctions (Flavia et al., 2006). This study showed that LCZ pretreatment could significantly mitigate the reduction in TEER caused by *E. coli*. This result corroborated the findings of several previous studies showing that *Lactobacillus* could significantly promote the TEER of Caco-2 and NCM460 cells (Horibe et al., 1997; Liu et al., 2010; Qiu et al., 2017). Accordingly, this study proposes that LCZ enhances the densification of monolayer BMECs. The same conclusion was obtained based on TEM observations of the desmosome structure between cells. Furthermore, tight junction proteins are known to maintain the structural integrity of tight junctions, which is necessary for the normal function of the blood-milk barrier (Shen et al., 2006; Guo et al., 2019). This study indicates that LCZ pretreatment could significantly increase tight junction protein expression in BMECs, which is consistent with the TEER results. These results are also consistent with those of Karczewski et al. (2010) and Wang et al. (2018b) showing that *Lactobacillus plantarum* could effectively promote occludin and ZO-1 expression in human intestinal cells as well as claudin-1 in IPEC-J2 cells. Moreover, Johnson et al. (2008) found that *Lactobacillus rhamnosus* could effectively promote claudin-1 and ZO-1 expression in T84 epithelial cells. The results of the Western blot, immunofluorescence, and the TEM *in vivo* test also showed that pre-infusion of LCZ could enhance the tight junction structure and upregulate tight junction protein expression in the mammary alveolar lumen in mice. This result is consistent with our *in vitro* test as well as previous studies, including Mennigen et al. (2009), Karczewski et al. (2010), and Patel et al. (2012), indicating that *Lactobacillus* can significantly promote the tightness of the intestinal epithelial barrier in both mice and humans by increasing tight junction protein expression. Consequently, this study found that LCZ could promote the integrity of the epithelial barrier *via* increasing tight junction protein expression in both BMECs and mice mammary tissues, and lead to lower expression of inflammatory cytokines, thereby alleviating damage to mammary tissue.

## Conclusion

This study shows that LCZ has prophylactic effect on *E. coli*-induced injury of mammary gland. It could counteract the blood-milk barrier disruption and decrease the inflammatory response, which mechanism included the promotion of tight junction protein expression and the inhibition of *E. coli* infection in both BMECs and mice mammary tissue. This study provides a new perspective on the protective effect of LCZ as well as an effective prophylactic agent for preserving blood-milk barrier function during *E. coli*-induced mastitis.

## Data Availability Statement

The original contributions presented in the study are included in the article/supplementary material, and further inquiries can be directed to the corresponding author.

## Ethics Statement

The animal study was reviewed and approved by the National Institutes of Health Guide for the Care and Use of Laboratory Animals (GB14925-2010, China).

## Author Contributions

YZ, GL, and SL conceived and designed the experiments. YZ performed the experiments, analyzed the data, and wrote the manuscript. GL, WW, and SL reviewed and edited the manuscript. GL, WW, YW, ZC, HY, and SL provided guidance for the experiments. All authors contributed to the article and approved the submitted version.

### Conflict of Interest

The authors declare that the research was conducted in the absence of any commercial or financial relationships that could be construed as a potential conflict of interest.
